# Modification of the BphP1-QPAS1 optogenetic system for gene expression regulation in Nicotiana benthamiana tobacco leaves using near-infrared light

**DOI:** 10.18699/vjgb-26-03

**Published:** 2026-03

**Authors:** E.S. Surkova, Y.A. Galimova, N.V. Battulina, D.M. Motorina, E.S. Omelina

**Affiliations:** Institute of Molecular and Cellular Biology of the Siberian Branch of the Russian Academy of Sciences, Novosibirsk, RussiaInstitute of Molecular and Cellular Biology of the Siberian Branch of the Russian Academy of Sciences, Novosibirsk, RussiaInstitute of Molecular and Cellular Biology of the Siberian Branch of the Russian Academy of Sciences, Novosibirsk, Russia Novosibirsk State University, Novosibirsk, Russia; Institute of Molecular and Cellular Biology of the Siberian Branch of the Russian Academy of Sciences, Novosibirsk, Russia; Institute of Molecular and Cellular Biology of the Siberian Branch of the Russian Academy of Sciences, Novosibirsk, Russia; Institute of Molecular and Cellular Biology of the Siberian Branch of the Russian Academy of Sciences, Novosibirsk, Russia; Institute of Molecular and Cellular Biology of the Siberian Branch of the Russian Academy of Sciences, Novosibirsk, RussiaInstitute of Molecular and Cellular Biology of the Siberian Branch of the Russian Academy of Sciences, Novosibirsk, Russia

**Keywords:** optogenetic system, BphP1-QPAS1, VVD, AsLOV2, Nicotiana benthamiana, gene expression regulation, Gal4/UAS, оптогенетическая система; ; ; ; ; ;, BphP1-QPAS1, VVD, AsLOV2, Nicotiana benthamiana, регуляция экспрессии генов, Gal4/UAS

## Abstract

In plants, the regulation of transgene transcription is typically achieved using chemical agents. A safe alternative to chemically induced systems may be optogenetic systems. The BphP1-QPAS1 system has distinct advantages over other optogenetic systems, as it is activated by near-infrared (NIR, 780 nm) light, which is beyond the spectrum of plant photoreceptors. This system is based on the use of a split transcription factor (TF), consisting of the DNA-binding and dimerization domains of the yeast TF Gal4, fused to the QPAS1 component, along with the transactivation domain VP16 fused to BphP1. Under NIR light, BphP1 interacts with QPAS1, leading to the formation of the functional TF Gal4-VP16. A primary obstacle to using optogenetic systems in plants is their undesired activation under white light, which is vital for normal plant growth. A potential solution to this issue is temporarily removing one component of the split TF from the nucleus under white light. We modified the BphP1-QPAS1 system to activate reporter gene expression in Nicotiana benthamiana leaves using NIR light. We combined BphP1-QPAS1 with several variants of LOV domain-containing proteins activated by blue light (460–480 nm). The best results were achieved by combining the BphP1-QPAS1 system with the AsLOV2 domain, which carries the degron sequence RRRG at the C-terminal Jα helix and initiates the degradation of the chimeric protein NES-Gal4-QPAS1-AsLOV2-RRRG under white light. This modification induced the BphP1-QPAS1 system in tobacco leaves only under NIR light, but not in the dark or under white light. We believe that, in the future, the BphP1-QPAS1 system could be applied to enhance plant resistance to adverse environmental conditions, pests, and viral diseases.

## Abstract (ru)

В растениях регуляция транскрипции трансгенов обычно осуществляется с помощью химических агентов. Безопасной альтернативой химически индуцируемым системам могут служить оптогенетические (т. е. светоиндуцируемые) системы. Бактериальная система BphP1-QPAS1 выгодно отличается от других оптогенетических систем, поскольку активируется ближним инфракрасным светом (БИК, 780 нм), выходящим за пределы спектра восприятия растительных фоторецепторов. Система BphP1-QPAS1 основана на использовании «расщепленного» транскрипционного фактора (ТФ), состоящего из ДНК-связывающего и димеризующего доменов дрожжевого ТФ Gal4, слитого с компонентом QPAS1, и трансактивационного домена VP16, слитого с BphP1. Под действием БИК света BphP1 взаимодействует с QPAS1, что приводит к сближению Gal4 с VP16 и запуску экспрессии репортерного гена. Основным препятствием для широкого использования оптогенетических систем в растениях является нежелательная активация таких систем при воздействии дневного (белого) света, который включает широкий спектр длин волн и жизненно важен для нормального роста растений. Решением проблемы нежелательной активации может стать временное удаление из ядра клетки одного из компонентов «расщепленного» ТФ при воздействии белого света. В данной работе мы модифицировали систему BphP1-QPAS1 для активации экспрессии репортерного гена в листьях табака Nicotiana benthamiana с помощью БИК света. Для этого мы комбинировали систему BphP1-QPAS1 с несколькими вариантами LOV домен-содержащих белков, активируемых синим светом (460–480 нм). Наилучшие результаты были достигнуты при комбинации компонентов системы BphP1-QPAS1 с доменом AsLOV2 из белка фототропин 1 Avena sativa, несущим на С концевой Jα спирали последовательность дегрона RRRG и запускающим деградацию химерного белка NES-Gal4-QPAS1-AsLOV2-RRRG при воздействии белого света. Такая модификация активировала систему BphP1-QPAS1 в листьях табака только при воздействии БИК света, но не в темноте либо при белом свете. Мы полагаем, что в будущем система BphP1- QPAS1 может быть применена для повышения устойчивости растений к неблагоприятным условиям среды, вредителям, вирусным заболеваниям.

## Introduction

The use of non-channel light-sensitive proteins, which can change conformation or form homo- or heterodimeric complexes under specific wavelengths of light, has enabled the application of optogenetic systems for precise temporal transcription regulation (Shimizu-Sato et al., 2002; Wang et al., 2012; Konermann et al., 2013; Ochoa-Fernandez et al., 2016; Gligorovski et al., 2023). Light control may serve as a valuable alternative to chemically inducible gene expression systems. The advantages of optogenetic systems over chemical ones include the following: (1) the ability to focus light on a specific small area of an organism or cell; (2) control over the intensity and duration of light exposure; (3) activation or deactivation of the system by switching the light On or Off, respectively; (4) independence from the diffusion rate of a chemical agent; and (5) absence of Off-target effects caused by chemical inducers (Shimizu-Sato et al., 2002; Omelina et al., 2022)

**Fig. 1.**
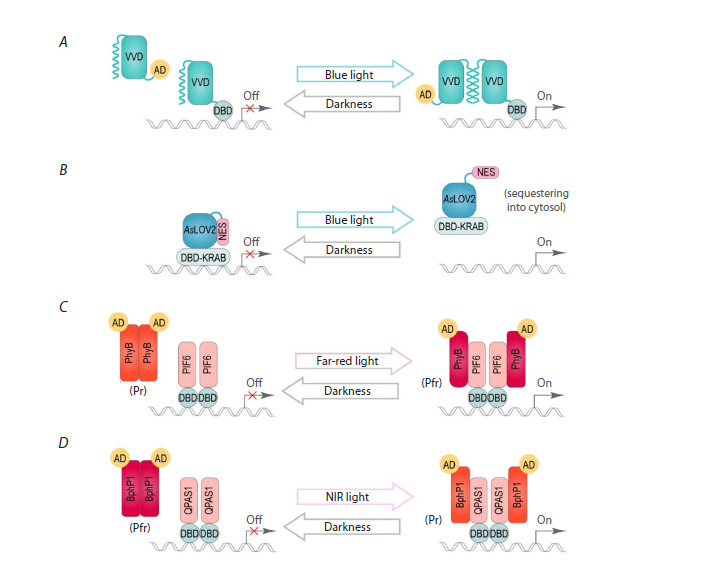
Different strategies for regulating gene expression using light. A, A schematic representation of the formation of homodimers due to conformational changes in the LOV domain of the VVD protein induced by blue light illumination. The N-terminal helix dissociates from the VVD core and interacts with the N-terminal helix of another VVD under blue light. As a result, the DBD and AD of the split TF associate into a functional TF, leading to the transcription of the reporter gene. B, A schematic representation of the unfolding of the C-terminal Jα helix from the AsLOV2 domain and the exposure of the fused NES signal, leading to the relocalization of the transcriptional repressor (DBD-KRAB) from the nucleus into the cytoplasm to control gene activity. C, D, A schematic representation of the formation of heterodimers PhyB-PIF6 under far-red light (C) or BphP1-QPAS1 under NIR light (D), and their dissociation in darkness. Upon light induction, the DBD interacts with the AD, resulting in the transcription of the reporter gene. The Pr and Pfr states refer to two interconvertible forms of phytochromes. AD, activating domain; DBD, DNAbinding domain; KRAB, Kruppel associated box; NES, nuclear export signal.

One of the strategies for regulating expression by light is based on photoreversible conformational changes (Fig. 1A, B). For instance, light illumination may induce the unfolding of one of the α-helices in the LOV domain-containing proteins (Crosson, Moffat, 2001; Zayner et al., 2012), which can be used to expose an engineered peptide that controls intracellular protein localization or protein-protein interactions (Krueger et al., 2019). Another strategy is the use of split transcription factors (TFs), where one component is a DNA-binding domain of a TF fused to a light-sensitive protein, while its partner is fused to an activator or repressor domain. When exposed to light, heterodimers are formed, resulting in the generation of an active transcription factor (Fig. 1C, D) (Pathak et al., 2017; Noda, Ozawa, 2018; Omelina, Pindyurin, 2019; Hernández- Candia, Tucker, 2020).

One of the most popular systems for the activation and suppression of gene transcription is the LOV domain-containing proteins. LOV domains belong to the Per-Arnt-Sim (PAS) protein family (Herrou, Crosson, 2011). The small size (~11– 15 kDa) and the presence of flavin chromophores in most, if not all, cell types are key advantages of these domains for the design of optogenetic tools (Wu Y.I. et al., 2009; Losi, Gärtner, 2011; Christie et al., 2012; Renicke et al., 2013; Niopek et al., 2016; Redchuk et al., 2017). One of the strategies for developing LOV-derived optogenetic tools is based on lightinduced homodimerization. This approach utilises the LOV domain-containing protein Vivid (VVD) from the filamentous fungus Neurospora crassa (Wang et al., 2012). VVD has an N-terminal helix that is docked on the core in the dark state. Blue light (460–480 nm) leads to the dissociation of this helix from the VVD core and interaction with the N-terminal helix of another VVD, resulting in the formation of a VVD homodimer (Fig. 1A) (Zoltowski et al., 2007; Zoltowski, Crane, 2008). The VVD-based strategy was used to develop the LightON system, providing blue-light-induced transcription of target transgenes in mammalian cells and in mice (Wang et al., 2012). VVD was fused with the DNA-binding domain of the yeast TF Gal4 and the p65 transcription activation domain. It formed homodimers under blue light, resulting in the generation of the active dimer Gal4-p65 and the transcriptional activation of the UAS reporter genes. Dark relaxation caused dimer dissociation, meaning the system returned to the non-active state.

Another LOV domain is derived from the LOV2 domain of Avena sativa phototropin 1 (AsLOV2). In LOV2, illumination with blue light leads to the unwinding of the caged C-terminal Jα helix and the exposure of the fused C-terminal peptide, thus making it available for protein-protein interactions (Peter et al., 2010; Lungu et al., 2012; Strickland et al., 2012; Yumerefendi et al., 2015; Kim et al., 2024; Kaya et al., 2025). This property of the LOV2 domain has been utilized to create a light-inducible nuclear export system (LEXY) (Niopek et al., 2016). In this system, blue light releases the photocaged nuclear export signal (NES) from the AsLOV2 core, thereby inducing the export of the protein of interest from the nucleus (Fig. 1B). To create this system, a library of 33 different NESs was generated, providing varying efficiencies of nuclear export for the chimeric protein NLS-mCherry-LEXY from the nucleus to the cytoplasm under blue light. This system has been tested in various mammalian cell cultures, and it has been demonstrated that during the dark relaxation period, the protein NLS-mCherry-LEXY returns to the nucleus, while the export of the protein from the nucleus can be repeatedly induced under blue light. An interesting application of LEXY is the spatiotemporal control of transcriptional repressors by sequestering them into the cytosol, which could be of particular value for controlling reporter gene activity (Fig. 1B) (Niopek et al., 2016).

Phytochromes are also a widely used optogenetic tool for the activation and suppression of gene transcription. These photoreceptors are found in plants, bacteria, cyanobacteria, and fungi, playing essential roles in light-adaptive processes. They share common domains in a photosensory core module and phytochrome-specific domains (Wagner et al., 2007; Yang et al., 2008; Chernov et al., 2017). Bacterial and fungal phytochromes incorporate a tetrapyrrole biliverdin IXα (BV) as a chromophore. In plants and cyanobacteria, BV is further enzymatically reduced to phytochromobilin (PΦB) or phycocyanobilin (PCB), which bind to plant and cyanobacterial phytochromes, respectively. Phytochromes exist in one of two interconvertible states: either the Pr state or the Pfr state (Fig. 1C, D). The Pr state absorbs light at 660–700 nm, whereas the Pfr state absorbs light at 740–780 nm (Li J. et al., 2011; Chernov et al., 2017). Typically, the ground (inactive) state of phytochromes is Pr, which converts to the active Pfr state upon illumination with 660–680 nm light (Fig. 1C) (Shcherbakova et al., 2015). A specific group of phytochromes, termed bathy, adopts Pfr as the ground state (Fig. 1D). Many photoreversible dimerization tools have been developed based on the light-regulated interaction of plant phytochrome with basic helix–loop–helix proteins known as phytochrome-interacting factors (PIFs). The plant phytochromes PhyA and PhyB from Arabidopsis thaliana are readily activated by far-red (640–680 nm) light and deactivated by near-infrared (NIR) (740–760 nm) light or in darkness. In the active Pfr state, PhyA and PhyB interact with PIF3 or PIF6 (Fig. 1C) (Ni et al., 1999; Zhu et al., 2000). A far-red light-inducible gene expression system in yeast was developed by fusing the components of the PhyB-PIF3 system to the DNA-binding and transactivation domains of the Gal4 TF (Shimizu-Sato et al., 2002). The interaction of PhyA and PhyB with PIF3 was exploited to develop a light-switchable gene expression system in mammalian cells (Müller et al., 2013). The PhyB-PIF6 system for activating transcription in plants has also been reported (Ochoa-Fernandez et al., 2020). PhyB is known to be very light-sensitive and can be easily activated even in dim white light. To prevent this, the authors combined the PhyB-PIF6 protein pair with a LOV-based optogenetic system. The system was successfully tested in tobacco and Arabidopsis

Among phytochromes, a subclass of bacterial phytochrome photoreceptors (BphPs) that incorporate BV should be emphasized (Giraud, Verméglio, 2008; Auldridge, Forest, 2011; Piatkevich et al., 2013). BV has the most red-shifted absorbance relative to other tetrapyrroles, including PΦB and PCB. The first BphP1-PpsR2 optogenetic system for transcriptional activation in eukaryotic cells was developed based on the bacterial bathy phytochrome BphP1 from the nonsulphur purple bacterium Rhodopseudomonas palustris. Under NIR light, BphP1 interacts with its natural partner protein PpsR2. This property was utilized to create a transcription activation system in mammalian cell cultures and mice (Kaberniuk et al., 2016). The original BphP1-PpsR2 system was optimized by reducing PpsR2 to a single-domain binding partner, QPAS1, which is three times smaller than full-length PpsR2, includes only the α-helical Q-linker and PAS1 domain and lacks oligomerization (Redchuk et al., 2017). The pair of proteins BphP1 and QPAS1 was used to create NIR light-induced systems for the activation (Fig. 1D) and suppression of gene expression, as well as for the control of protein localization. These systems were tested in various mammalian cell cultures (Redchuk et al., 2017, 2018).

Previously, optogenetic systems for the activation of expression in plants utilized the plant phytochrome PhyB (Müller et al., 2014; Ochoa-Fernandez et al., 2016, 2020) or the bacterial photoreceptor CarH (Chatelle et al., 2018) activated by far-red (640–680 nm) or green light (525 nm), respectively (Omelina et al., 2022). A significant drawback of these systems is the potential effect on endogenous plant photoreceptors (Müller et al., 2014; Battle, Jones, 2020; Christie, Zurbriggen, 2021). In this study, for the regulation of the reporter gene transcription in plants, we applied the bacterial BphP1-QPAS1 system activated by NIR light (780 nm), which is outside the spectra of plant photoreceptors’ excitation and differs from the wavelength required for their reversion. BphP1 is known to incorporate BV as a chromophore, which is synthesized in chloroplasts as a precursor of PΦB, suggesting the usability of BV-required tools in plants (Shikata, Denninger, 2022). However, the application of optogenetic systems in plant research is known to have a significant obstacle. Plants are phototrophic organisms and require exposure to white light, which may nonspecifically activate optogenetic systems (Andres et al., 2019). To block the activity of the BphP1-QPAS1 system under white light, we combined the BphP1-QPAS1 protein pair with blue light-controlled systems (the LOV domain-containing VVD protein or AsLOV2 domain fused to several variants of the NES and degron sequences). The best results were observed for the combination of the BphP1-QPAS1 system with the AsLOV2 domain fused to a degron sequence. In the leaves of these transiently transformed tobacco plants, we observed strong fluorescence of the plant EGFP (pEGFP) reporter under NIR light and found no pEGFP signal when the plants were incubated in standard day/night conditions

We believe that in the future, the optogenetic system BphP1- QPAS1 will enable the development of several approaches for creating safe and highly productive agricultural plants that are resistant to stress conditions, pests, and viral diseases, which are common among agricultural crops. Unlike methods that use various chemicals to combat pests and infectious diseases, optogenetic biotechnologies are non-toxic and facilitate safe and high-quality food production.

## Materials and methods

Design of plasmids. All plasmids generated in this study are presented in the Table

**Table 1.**
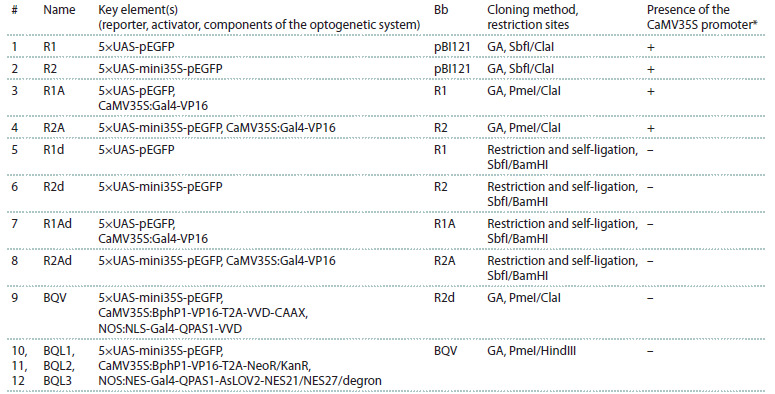
Plasmids for testing the Gal4/UAS (#1–8) and optogenetic (#9–12) systems in tobacco leaves Note. Bb, backbone; GA, Gibson assembly method. * The full-length promoter CaMV35S from the pBI121 vector backbone, which controls expression of the GUS reporter gene.

**Fig. 2.**
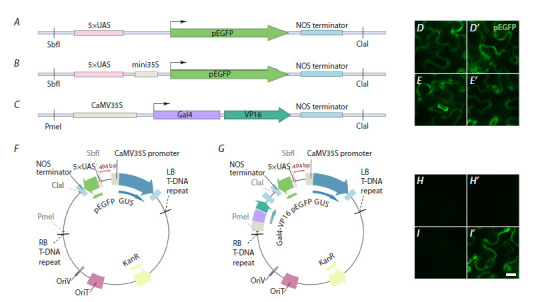
Structure of the control reporter plasmids for testing the Gal4/UAS system in transiently transformed N. benthamiana leaves. A, Reporter construct R1 without a minimal promoter. B, Reporter construct R2, containing the sequence of the minimal CaMV35S promoter – mini35S. C, Scheme of the light-independent Gal4-VP16 activator, incorporated into the reporter plasmids R1 and R2 for the activation of the reporter gene. It consists of the DNA-binding and dimerization domains of the yeast TF Gal4 (148 a. a.) fused with the transactivation domain VP16. The plasmid elements are not shown to scale. D–E ’ , Microscopy images of N. benthamiana leaves transiently transformed with the constructs R1 (D), R1A (D ’ ), R2 (E), R2A (E ’ ). F, A scheme of the reporter construct R1 (analogous to R2) based on the pBI121 vector backbone. G, A scheme of the construct R1A (analogous to R2A) with the Gal4-VP16 activator based on the pBI121 vector backbone. F, G, The full-length CaMV35S promoter, which regulates the activity of the GUS gene, is located 494 bp from the UAS repeats in the reverse orientation. H–I ’ , Microscopy images of N. benthamiana leaves transiently transformed with the constructs R1d (H), R1Ad (H ’ ), R2d (I), R2Ad (I ’ ). Plants were incubated in simulated day/night conditions for 48 hours (D–E ’ , H–I ’ ). Scale, 20 μm.

Control reporter plasmids without the activator (R1, R2). Two variants of reporter plasmids R1 and R2 (Fig. 2A, B; Supplementary Figs. 1, 2)1 were obtained using the Gibson assembly method based on the pBI121 vector (Chen et al., 2003), which was hydrolyzed at the SbfI/ClaI restriction sites. For that, the UAS sequence was amplified from the pUASTattB vector (GenBank EF362409.1) (Bischof et al., 2007). The pEGFP coding sequence, fused with the NOS terminator, and the sequence of the minimal promoter from the Cauliflower mosaic virus (mini35S) were amplified from the pCAMBIA_pGFP vector (Gayatri, Basu, 2020).

Supplementary Materials are available in the online version of the paper: https://vavilovj-icg.ru/download/pict-2026-30/appx5.zip


Control reporter plasmids with the activator (R1A, R2A). The sequences of the full-length promoter of the Cauliflower mosaic virus (CaMV35S) and light-independent activator Gal4-VP16 were added to the R1 and R2 plasmids to obtain the R1A and R2A plasmids. The CaMV35S promoter sequence was amplified from the pBI121 vector. The sequence of the activator Gal4-VP16 (Fig. 2C; Suppl. Fig. 3) was amplified from the plasmid pGal4-VP16 (Redchuk et al., 2017). The NOS terminator sequence was amplified from the pCAMBIA_pGFP vector. All fragments were inserted into the reporter plasmids R1 or R2 using the Gibson assembly method after being hydrolyzed at the PmeI/ClaI restriction sitesControl reporter plasmids without the full-length CaMV35S promoter from the pBI121 plasmid. To eliminate the influence of the CaMV35S promoter from the pBI121 plasmid on the expression of pEGFP, this fragment was excised from R1, R2, R1A and R2A plasmids at the SbfI/BamHI sites. Subsequently, the vectors were treated with a Klenow fragment (NEB), followed by self-ligation. These constructs were named R1d, R2d, R1Ad and R2Ad, respectively

**Fig. 3.**
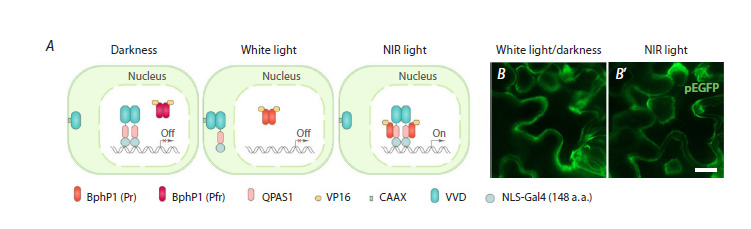
NIR light-controlled transcription activation of the reporter gene using the BphP1-QPAS1 system in combination with the VVD protein A, A scheme of the proposed functioning of the BphP1-QPAS1 system under different light conditions. In darkness, BphP1 exists in the inactive Pfr state and cannot interact with QPAS1. Under white light, BphP1 converts to the active Pr state. However, it cannot interact with QPAS1, as the NLS-Gal4-QPAS1-VVD chimeric protein relocalizes to the plasma membrane due to dimer formation with VVD-CAAX. Exposure to NIR light leads to the interaction of the BphP1 and QPAS1 components in the nucleus and activation of the reporter gene expression. B, B ’ , Microscopy images of N. benthamiana leaves transiently transformed with the plasmid BQV. The plants were incubated in simulated day/night conditions for 72 hours (B) or in simulated day/night conditions for 48 hours, followed by incubation under NIR light for 24 hours (B ’ ). Scale, 20 μm.

Plasmid carrying the coding sequences of the BphP1-VP16, NLS-Gal4-QPAS1-VVD and VVD-CAAX chimeric proteins (BQV). To generate the BQV plasmid (Suppl. Fig. 4), the components of the BphP1-QPAS1 system were amplified from the plasmid pQP-T2A (Addgene #102583 (Redchuk et al., 2017)). The coding sequence of the VVD protein was amplified from the GAVPO construct (Wang et al., 2012). The NLS and CAAX sequences were included in the primers. These fragments were inserted into the reporter plasmid R2d using the Gibson assembly method after being hydrolyzed at the PmeI/ClaI restriction sites.

**Fig. 4.**
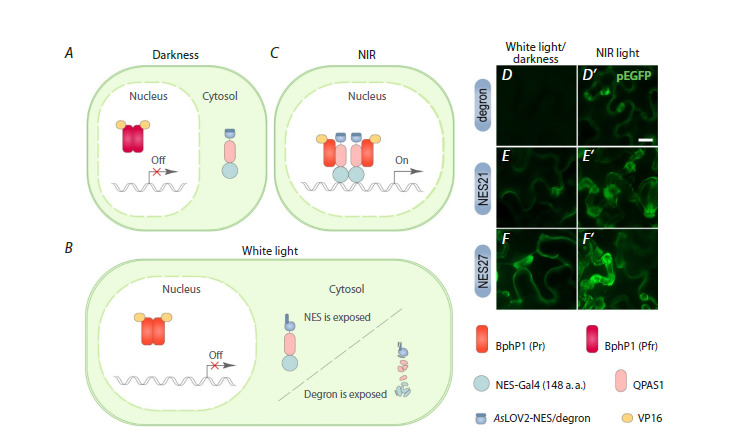
Two strategies of the BphP1-QPAS1 system functioning to avoid undesired activation in simulated day/night conditions. A, A schematic representation of the proposed functioning of the modified BphP1-QPAS1 system in darkness. BphP1-VP16 exists in the ground Pfr state and predominantly resides in the nucleus of the cells. The NES-Gal4-QPAS1-AsLOV2-NES/degron localizes in the cytoplasm due to a weak N-terminal NES signal. B, A scheme of the proposed functioning of the modified BphP1-QPAS1 system under white light. BphP1-VP16 possibly exists in the active Pr state. However, it cannot interact with QPAS1, as under white light, the NES or degron signal unwinds from the AsLOV2 core. This results in the relocalization from the nucleus to the cytoplasm or degradation of the NES-Gal4-QPAS1-AsLOV2-NES/degron chimeric protein. C, A scheme of the proposed functioning of the modified BphP1-QPAS1 system under NIR light. BphP1 exists in the active Pr state and interacts with QPAS1, leading to the formation of the functional TF Gal4-VP16 and the activation of the reporter gene expression. D–F ’ , Microscopy images of N. benthamiana leaves transiently transformed with plasmids carrying the components of the BphP1-QPAS1 system in combination with AsLOV2 fused to different peptides (degron in D, D ’ ; NES21 in E, E ’ ; NES27 in F, F ’ ). The plants were incubated in simulated day/night conditions for 72 hours (D, E, F) or in simulated day/night conditions for 48 hours, followed by incubation under NIR light for 24 hours (D ’ , E ’ , F ’ ). Scale bar, 20 μm.

Plasmids carrying the coding sequences of the BphP1-VP16 and NES-Gal4-QPAS1-AsLOV2-NES21 or NES-Gal4-QPAS1- AsLOV2-NES27 or NES-Gal4-QPAS1-AsLOV2-RRRG chimeric proteins (BQL1, BQL2, BQL3, respectively). To generate the BQL1, BQL2 and BQL3 plasmids (Suppl. Fig. 5), the sequences of the NOS and CaMV35S promoters, the NOS terminator, and the NeoR/KanR sequence were amplified from the pBI121 vector. The components of the BphP1-QPAS1 system were amplified from the plasmid pQP-T2A. The Cauliflower mosaic virus polyadenylation signal sequence was amplified from the pCAMBIA_pGFP vector. The AsLOV2

Preparation of chemically competent Agrobacterium tumefaciens cells. An overnight culture (3 ml) of A. tumefaciens strain GV3101 was grown in sterile YEP medium (10 g/l yeast extract, 10 g/l Bacto Peptone, 5 g/l NaCl) for 16–20 hours at 28 °C. Subsequently, 2 ml of this culture was transferred to a flask containing 50 ml of sterile YEP medium and incubated at 28 °C until an optical density at 600 nm (OD600) of 0.5–1.0 was reached (typically several hours to overnight). The culture was then cooled on ice for 30 minutes. Cells were harvested by centrifugation at 5,000g at 4 °C for 8 minutes. The supernatant was discarded, and the cell pellet was gently resuspended in 2 ml of ice-cold 20 mM CaCl2. The suspension was incubated on ice for an additional 30 minutes. Aliquots of 100 μl were dispensed into pre-chilled microcentrifuge tubes, flash-frozen in liquid nitrogen, and stored at –70 °C.

A. tumefaciens transformation. Chemically competent A. tumefaciens strain GV3101 was transformed with the plasmids of interest (see the Table). Specifically, 1 μg of plasmid DNA was added to 100 μl of competent cells. These cells were thawed for 5 minutes at 37 °C, after which 1 ml of YEP medium supplemented with 50 μg/ml kanamycin and 100 μg/ml rifampicin was added. The mixture was incubated with shaking for 3 to 5.5 hours at 28 °C. The cells were then centrifuged at 1,000g for 5 minutes, after which the pellet was resuspended in 100 μl of YEP medium and plated on YEP agar plates containing kanamycin and rifampicin. Plates were incubated in an inverted position at 28 °C for 72 hours until individual colonies appeared. Colonies were confirmed by colony PCR using Taq DNA polymerase (Biolabmix).

Transient transformation of Nicotiana benthamiana plants. A. tumefaciens cultures were adjusted to an optical density measured at a wavelength of 600 nm (OD600 = 0.5) in infiltration medium (10 mM MgCl2, 10 mM MES in H2O). The cultures were incubated for 1–3 hours at 28 °C prior to infiltration through the adaxial surface of the leaves of 4- to 5-week-old N. benthamiana

Illumination conditions and microscopy. To test the Gal4/ UAS system (plasmids #1–8 in the Table) in tobacco leaves, the plants were kept in a plant incubator with fluorescent tubes (cool white light, OSRAM), employing alternating cycles of light and darkness (16 hours of light and 8 hours of darkness) to simulate standard day/night conditions for 48 hours

To analyze the modified BphP1-QPAS1 system variants, N. benthamiana plants transiently transformed with the plasmids BQV, BQL1, BQL2, or BQL3 (see the Table) were grown for 48 hours in simulated day/night conditions (16 hours of white light and 8 hours of darkness). Thereafter, half of the plants were incubated under NIR light for 24 hours to induce pEGFP expression, while the other half continued to grow under the same day/night conditions NIR illumination was performed using custom-assembled LED arrays with a wavelength of 780/20 nm. For microscopic analysis, we conducted at least three independent experiments, taking 1–3 samples from different areas of the tobacco leaves and using a Zeiss Axio Imager M2 (Carl Zeiss).

## Results

**Testing of light-independent expression of the control reporter constructs in tobacco leaves**

To test the functioning of the optogenetic system BphP1- QPAS1 in plants, we first selected an optimal structure for the regulatory region of the UAS enhancer-based reporter construct. This structure should provide minimal reporter activity in the absence of the activator and a high level of reporter gene expression in the presence of the activator. The creation of different variants of the reporter construct for use in N. benthamiana is necessary due to the lack of a single version of the UAS regulatory regions of reporter genes in plants, as their structure depends on the plant species (Wu C. et al., 2003; Johnson A.A.T. et al., 2005; Sakvarelidze et al., 2007; Yun et al., 2023). Additionally, the DNA-binding activity of Gal4 is known to be sensitive to the methylation of its binding site in plant chromatin, which can result in weak expression or even the loss of Gal4-mediated expression (Gälweiler et al., 2000). To develop these control reporter constructs, we employed the widely used binary Agrobacterium vector pBI121, which carries a GUS (β-glucuronidase) reporter gene for plant transformation (Chen et al., 2003). Based on the pBI121 vector backbone, we designed two variants of the control reporter plasmids, R1 (Fig. 2A; the Table; Suppl. Fig. 1) and R2 (Fig. 2B; the Table; Suppl. Fig. 2). Both plasmids contain five UAS repeats for the binding of the TF Gal4-VP16 and the reporter gene pEGFP. The R2 plasmid additionally contains the sequence of the minimal promoter from the Cauliflower mosaic virus (CaMV35S) – mini35S.

The sequence of the light-insensitive Gal4-VP16 activator was added to the reporter plasmids R1 and R2, resulting in the creation of the R1A and R2A plasmids (see the Table). The Gal4-VP16 activator consists of the DNA-binding and dimerization domains of the yeast TF Gal4 (148 a. a.) fused with the transactivation domain VP16 (Fig. 2C; Suppl. Fig. 3). For testing in plants, the plasmids R1, R2, R1A, and R2A were used to transform A. tumefaciens cells, which were subsequently used to infiltrate the leaves of the tobacco N. benthamiana. A microscopic analysis of pEGFP protein expression in the transiently transformed tobacco leaves was conducted 48 hours after infiltration. We anticipated observing pEGFP expression in the tobacco leaves transformed with one of the variants of the reporter plasmids in the presence of the Gal4-VP16 activator (R1A or R2A), but not in its absence (R1 or R2) (see the Table). However, all the analyzed constructs exhibited strong pEGFP expression (Fig. 2D–E′).

As we mentioned above, we cloned the reporter plasmids based on the pBI121 vector backbone. This vector carries the GUS coding sequence under the control of the full-length CaMV35S promoter located 494 bp from the UAS repeats in the reverse orientation (Fig. 2F, G). We did not initially remove the GUS sequence and its promoter from the vector, assuming that its expression would not interfere with the analysis of the pEGFP fluorescence. However, a thorough review of the literature indicated that the full-length CaMV35S promoter could alter the activity level of adjacent genes located approximately 2 kb downstream of the promoter (Zheng et al., 2007). Furthermore, this promoter can act as an enhancer in either orientation and lead to an increase in the expression level of neighboring genes (Zheng et al., 2007). To eliminate the presumed influence of the CaMV35S promoter on the expression of the pEGFP reporter, we removed its sequence from plasmids R1, R2, R1A and R2A. As a result, we obtained two new variants of the reporter constructs (R1d and R2d) and two constructs that carry both the reporter gene pEGFP and the activator Gal4-VP16 (R1Ad and R2Ad) (see the Table). Analysis of the activity of these constructs in tobacco leaves showed that pEGFP protein expression was only observed in the leaves transformed with the R2Ad construct with the reporter containing the mini35S sequence in the presence of the Gal4-VP16 activator (Fig. 2I ′). In the case of the other constructs, the level of the pEGFP signal corresponded to autofluorescence (Fig. 2H–I ). Therefore, we subsequently used the R2d reporter plasmid for the application of the optogenetic system BphP1-QPAS1 in plants.

**Optogenetic control of gene expression with the BphP1-QPAS1 system in transiently transformed N. benthamiana leaves**The application of optogenetic systems in plant research is known to face a significant obstacle in the form of undesired activity under ambient white light, which is vital for normal plant growth and development (Braguy, Zurbriggen, 2016). The original BphP1-QPAS1 system is based on the split TF Gal4-VP16, which is assembled by exposure to NIR light. The DNA-binding domain of Gal4 is fused to QPAS1 (Gal4- QPAS1) and BphP1 is fused with the VP16 transactivation domain (BphP1-VP16) (Fig. 1D) (Redchuk et al., 2017). To address the problem of undesired activation, we fused the Gal4-QPAS1 chimeric protein with several variants of blue light-sensitive LOV domains. This approach might provide the removal of the Gal4-QPAS1 component from the cell nucleus under white light.

We combined the components of the BphP1-QPAS1 system with the VVD protein (see the BQV plasmid in the Table), which forms homodimers under blue light (or white light covering a broad spectrum of different wavelengths, including blue light at 460–480 nm). To achieve this, we fused the DNA-binding protein NLS-Gal4-QPAS1 with VVD, resulting in the chimeric protein NLS-Gal4-QPAS1-VVD (Fig. 3A). We also added to the BQV plasmid an additional VVD-containing construct, VVD-CAAX, which facilitates the attachment of the VVD protein to the plasma membrane via the CAAX peptide (Fig. 3A; Suppl. Fig. 4) (Redchuk et al., 2017; Willumsen et al., 1984a, b). We hypothesized that in this modified variant of the optogenetic system, BphP1-VP16 would predominantly reside in the nucleus of the cells (Fig. 3A). In darkness, the system exists in a state of relaxation. Under white light, the chimeric proteins NLS-Gal4-QPAS1-VVD and VVD-CAAX form dimers due to VVD homodimerization, resulting in the relocalization of the chimeric protein NLS-Gal4-QPAS1-VVD to the membrane. Under NIR light, BphP1 interacts with QPAS1, leading to the activation of the reporter gene (Fig. 3A).

To test the functionality of the BphP1-QPAS1 system combined with VVD, we transformed Agrobacterium with the BQV plasmid (see the Table), followed by infiltration of the leaves of tobacco plants. The plants were incubated in simulated day/night conditions (16 hours of light and 8 hours of darkness) for 48 hours. Thereafter, half of the plants were incubated under NIR light for 24 hours, while the other plants continued to grow in simulated day/night conditions. As a result, we detected pEGFP fluorescence in the transiently transformed tobacco leaves incubated both under NIR light and in white light/dark conditions (Fig. 3B, B′).

It should be noted that a standard practice for testing optogenetic systems involves incubating samples under different light conditions: in darkness and under pulsed light (e. g., 30 seconds On and 180 seconds Off). Initially, after the transient transformation of the tobacco leaves with the BQV plasmid, we employed various light conditions. We kept the plants either in darkness or under continuous blue (480 nm), NIR (780 nm), or white light for 48 hours. However, these conditions were not optimal for the normal growth of the plants, and they exhibited wilting. Therefore, in all subsequent experiments, we decided to simulate the most natural lighting conditions with a day/night cycle during the first 48 hours after transformation. Thereafter, half of the plants were incubated under NIR light for 24 hours to induce pEGFP expression, while the remaining plants continued to grow in simulated day/night conditions.Thus, the combination of BphP1-QPAS1 with the used variant of the VVD protein appears to be ineffective in plants, as the addition of VVD did not eliminate the undesired activity of the BphP1-QPAS1 system under white light (and possibly in darkness). To address this issue, we first replaced the N-terminal nuclear localization signal (NLS) with a weak NES signal in the Gal4-QPAS1 component in the BQV plasmid. It may relocalize part of this chimeric protein from the cell nucleus, which could potentially reduce the level of undesired activation in darkness. Secondly, we replaced VVD with the blue-light-sensitive AsLOV2 domain, which carries a specific peptide on its C-terminal Jα helix. Utilizing the different peptides allowed for two strategies: (1) white light-induced nuclear export of the chimeric protein NES-Gal4-QPAS1-AsLOV2- NES; (2) white light-induced degradation of the chimeric protein NES-Gal4-QPAS1-AsLOV2-degron (Fig. 4A–C). To relocalize the NES-Gal4-QPAS1-AsLOV2-NES chimeric protein from the nucleus to the cytosol, we used two different NES signals, presented in plasmids BQL1 and BQL2 (see the Table; Suppl. Fig. 5). These signals (NES21 and NES27, respectively) were taken from a library of NES signals, which, in fusion with AsLOV2, have shown different efficiencies of nucleocytoplasmic translocation under blue light (Niopek et al., 2016). For light-inducible degradation of the NES-Gal4- QPAS1-AsLOV2-degron protein, the plasmid BQL3 was generated (see the Table; Suppl. Fig. 5). For this strategy, we utilized the previously described B-LID system carrying the degron variant RRRG (Bonger et al., 2014)

We transformed Agrobacterium with the plasmids BQL1, BQL2, and BQL3, followed by the infiltration of tobacco leaves. The plants were incubated for 48 hours in simulated day/night conditions. Thereafter, half the plants were incubated under NIR light for 24 hours, while the others continued to grow in simulated day/night conditions for a further 24 hours. The strategy based on white light-sensitive degradation enabled the avoidance of undesired activation of the BphP1- QPAS1 system under white light and in darkness. We observed a strong pEGFP signal under NIR light and did not detect any pEGFP fluorescence in plants grown under standard day/ night conditions (Fig. 4D, D′). The strategy based on white light-induced nucleocytoplasmic translocation was much less effective. Both variants of the NES signals displayed pEGFP fluorescence under NIR light as well as in alternating cycles of white light and darkness (Fig. 4E–F ′).

## Discussion

The undesired activation of optogenetic systems under ambient white light is known to be a significant obstacle to using these systems in plant research, as white light is vital for normal plant growth. This explains the relatively low number of articles on the application of optogenetic systems in plants compared to studies employing chemically inducible systems (Omelina et al., 2022). However, optogenetic systems offer several undeniable advantages over chemical systems, making their application in plants very appealing. To address the problem of undesired activation, optogenetic systems may be combined with other light-sensitive proteins that have higher light sensitivity to block undesired activity under white light.In this study, we first applied the BphP1-QPAS1 system in the leaves of N. benthamiana to induce the expression of a reporter gene using NIR light. To avoid undesired activity of the BphP1-QPAS1 system under white light in plants, we combined it with the blue light-sensitive VVD protein (Wang et al., 2012). Previous studies in mice have demonstrated that VVD is more light-sensitive compared to the BphP1-QPAS1 protein pair (Kaberniuk et al., 2016). However, in plants, the combination of these systems did not yield the desired result. This may be related to the previously described dependence of the photoinducible dimerization properties of VVD on temperature (Qian et al., 2023). In this study, we used a variant of the VVD protein bearing the mutations N56K and C71V, which have previously shown a high On/Off ratio at 37 °C and a low On/Off ratio at 25 °C in HEK293 cells (Qian et al., 2023). The combination of the BphP1-QPAS1 system with the VVD protein is very appealing for use in plants, as all components are orthogonal to plant cells. Using another mutant version of the VVD protein or other localization signals in the future might enable avoiding undesired activation of the BphP1- QPAS1 system in combination with VVD under white light. We replaced the VVD protein with the plant-derived AsLOV2 domain. As a result, a variant of the AsLOV2 domain fused with the degron sequence has shown very good results. We observed a high level of pEGFP fluorescence after NIR light illumination and did not detect the pEGFP signal in plants grown in simulated day/night conditions.

It is important to note that light-sensitive proteins of plant origin have been applied in plants before. For instance, the PULSE system, which was previously successfully tested in plants, is based on the use of the plant phytochrome PhyB (Müller et al., 2014; Ochoa-Fernandez et al., 2020). In this system, the problem of undesired activation under white light was solved by the addition of the bacterial LOV-domain protein EL222 fused with the repressor domain SRDX and binding to the same promoter of the reporter gene as the PhyB-PIF6-based split TF. We propose that the BphP1-QPAS1 system has several advantages compared to the PhyB-based system, as its activation occurs at NIR light (780 nm) rather than at 640–680 nm. NIR light is preferable not only because it is not toxic to eukaryotic cells, including plant cells, but also because it does not affect the excitation or relaxation of endogenous phytochromes (Johnson M.P., 2016; Guidi et al., 2017). Additionally, passive activation of degradation by white light (but not blue light) does not affect the activation of endogenous phototropins and cryptochromes. The chromophores required for photoactivation of BphP1 and AsLOV2 (BV and flavin chromophore, respectively) are plant metabolites and are present in sufficient quantities in plant cells. In contrast to BphP1-QPAS1, the functioning of other optogenetic systems may lead to the potential activation of endogenous photoreceptors, which play a crucial role in plant development. Moreover, it should also be noted that the BphP1-based system is significantly less sensitive to ambient light compared to PhyB-derived optogenetic tools (Kaberniuk et al., 2016). Furthermore, the PhyB protein is present in all seed plants (Mathews, 2010; Li F.W. et al., 2015), so the interaction of the components of PhyB-based systems with endogenous proteins cannot be ruled out. These points favourably distinguish the BphP1-QPAS1 system from previously used systems for light-inducible regulation of gene expression in plants. Thus, in the near future, the BphP1-QPAS1 system has all the potential for wide application in plant research and biotechnology, for example, for plant protection from adverse climatic and chemical conditions (such as drought, toxins, soil salinity, and radiation), plant diseases (including moulds, bacteria, and viruses), and pests (such as insects and rodents).

## Conflict of interest

The authors declare no conflict of interest.
